# Cyclohexane, a Potential Drug of Abuse with Pernicious Effects on the Brain

**DOI:** 10.3389/fphar.2015.00291

**Published:** 2016-01-07

**Authors:** Tania Campos-Ordonez, Oscar Gonzalez-Perez

**Affiliations:** Laboratory of Neuroscience, School of Psychology, University of ColimaColima, Mexico

**Keywords:** oxidative stress, organic solvents, APE-1, neurotoxicity, astrocyte, microglia, toluene, inhalant abuse

## Abstract

Cyclohexane is a volatile solvent used as a harmless substitute for dangerous organic solvents in several products, such as paint thinners, gasoline and adhesives. Many of these products are used as drugs of abuse and can severely damage neural tissue and impair neurological functions. However, there is very little information on the effects of cyclohexane on the brain. In humans, cyclohexane produces headaches, sleepiness, dizziness, limb weakness, motor changes, and verbal memory impairment. Recent studies in mice have demonstrated behavioral alterations, reactive gliosis, microglial reactivity, and oxidative stress in the brains of cyclohexane-exposed animals. This indicates that cyclohexane may represent a potential problem for public health. Therefore, studies are needed to clarify the neurobiological effects of this volatile compound, including the cellular and molecular mechanisms of neurotoxicity, and to minimize the human health risk posed by the intentional or accidental inhalation of this potential drug of abuse.

Inhalants are used as drugs of abuse by a large number of people globally. These substances are found in numerous inexpensive and legally available commercial (thinners, gasoline, and adhesives, etc.), which are widely available at supermarkets, workplaces, and online (Ridenour et al., 2007). In the United States of America, approximately 5.2% of teenagers reported inhalant use at least once in their lifetime (Johnston et al., 2014). The volatile compounds can be inhaled by various methods, which are referred to as “sniffing,” “snorting,” “huffing,” and “bagging.” Typically, the duration of inhalation is a few minutes (10–15 min). However, during this period, a high concentration of solvents (above 6000 ppm) can be inhaled, and this routine may be performed several times a day (Bowen et al., 2006).

Inhalation of solvents has pernicious effects on the brain, produces severe systemic impairments, and increases the risk of suicide and death (Ridenour et al., 2007). Solvent abuse can result in neurological disorders, including psychiatric diseases such as depression, anxiety, bipolar mood disorder, and addiction (Ridenour et al., 2007). The long-term exposure to organic solvents may also produce chronic encephalopathy, which is characterized by abnormalities in brain structures and cognitive dysfunction (Ramcharan et al., 2014).

Usually, commercial solvent abuse results in exposure to several volatile substances, such as toluene, *n*-hexane, xylene and benzene. This makes it difficult to study the neurotoxic effects of the individual constituents (Ramcharan et al., 2014). Therefore, researchers have to study the effects of each solvent to clarify their role in brain degeneration and neurological impairment.

Cyclohexane is a volatile substance that has been implicated in cognitive deterioration (Bespalov et al., 2003; Lammers et al., 2009). Initially, cyclohexane was considered a safe substitute for benzene and toluene because of its lack of carcinogenic effects and low toxicity (Sikkema et al., 1995; Yuasa et al., 1996). However, cyclohexane is a strongly lipophilic molecule that can easily diffuse through neural tissue and target numerous brain regions (Figure 1). The effect of cyclohexane inhalation on the nervous system was first evaluated in shoe workers. After a 6-h exposure to low levels of this solvent, subjects develop dimmed vision (Yasugi et al., 1994), sleepiness, dizziness, limb weakness, sensorial disturbances (hypoesthesia and paresthesia), and motor dysfunction of the median, ulnar and peroneal nerves (Mutti et al., 1982; Yuasa et al., 1996). Volunteers exposed to a moderate concentration of cyclohexane (250 ppm) reported a higher incidence of headache, dry throat and verbal memory impairment than subjects exposed to very low concentrations of the compound (25 ppm; Lammers et al., 2009). In this study by Lammers et al. (2009), the cyclohexane concentrations corresponded to typical occupational exposure levels. However, the effects of recreational doses of cyclohexane (often above 6000 ppm) remain unknown. Identifying the minimum concentration of cyclohexane that produces neural degeneration would help regulators set limits on the concentration of this solvent in commercially available products.

**Figure 1:**
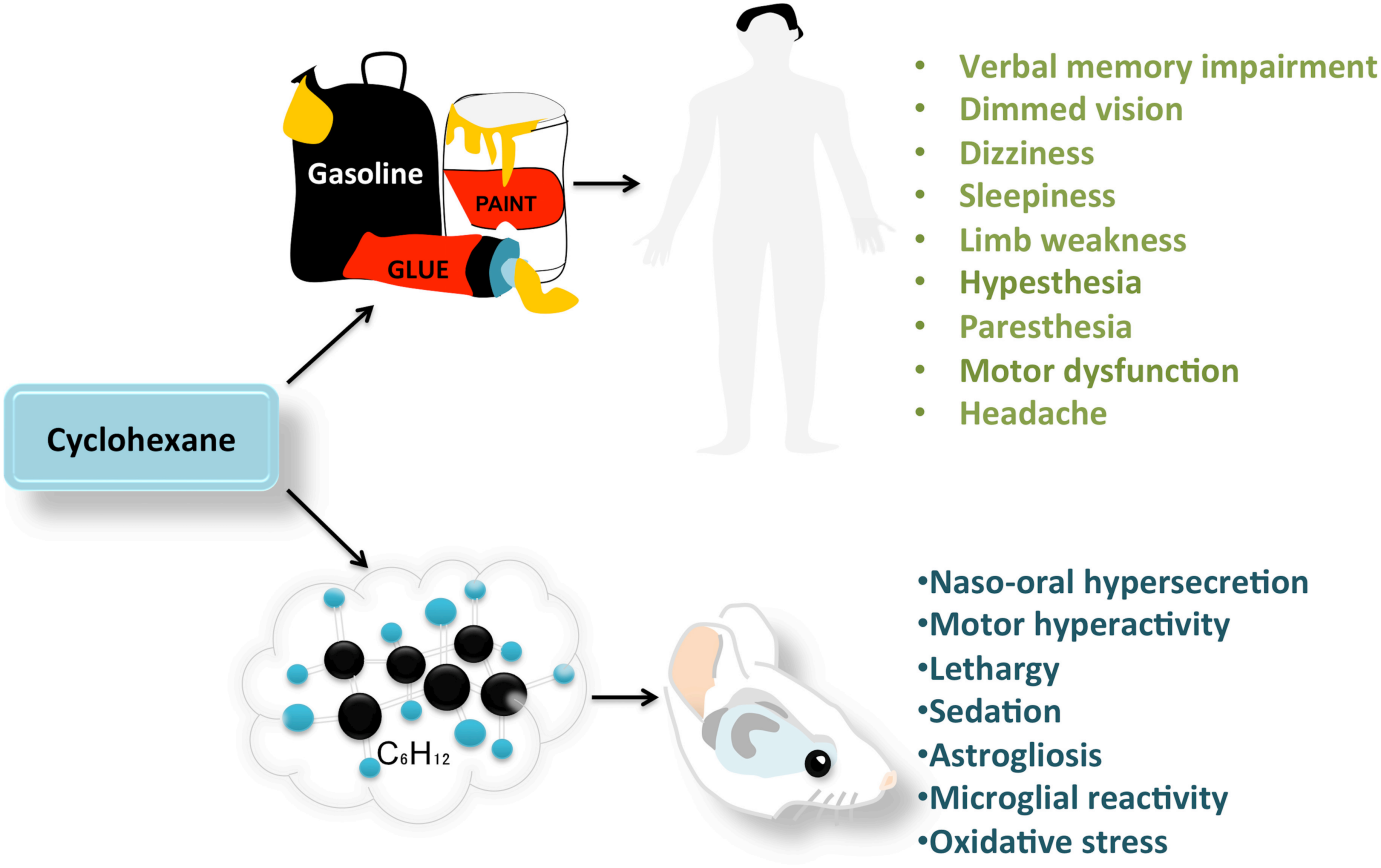
**Behavioral and histological changes observed after cyclohexane inhalation in humans and rodents**.

The clinical features of solvent abusers include motor impairment, euphoria, excitability, ataxia and depression. Solvents such as toluene and trichloroethylene (TCE) exhibit biphasic dose-response curves, characterized by motor excitation at low exposure levels, and motor impairment, sedation and anesthesia at high exposure levels (Bowen et al., 2006). Interestingly, a similar biphasic dose-response effect has been described in mice exposed to cyclohexane, and is associated with neurohistological changes (Campos-Ordonez et al., 2015). Furthermore, these volatile solvents produce dramatic structural changes in the brain, including atrophy of the cerebral cortex, white matter, corpus callosum, hippocampus, brainstem, cerebellum, basal ganglia, red nuclei, and substantia nigra (Fan et al., 2014; Ramcharan et al., 2014). Experimental models of toluene, 1-bromopropane, TCE and dichloromethane exposure have revealed the presence of astrocyte reactivity and a microglial response in the hippocampus, cerebellum, and cerebral cortex. The astroglial response to brain insults is characterized by increased cell proliferation, hypertrophy, and increased expression of glial fibrillary acidic protein (GFAP; Gonzalez-Perez et al., 2015). In comparison, the microglial response is characterized by dramatic morphological changes that include a transition to an amoeboid morphology and a reduction in cellular processes (Gonzalez-Perez et al., 2012).

Cyclohexane at concentrations typical of those used by recreational drug users (9000 ppm) also induces a glial cell response in the hippocampus (Campos-Ordonez et al., 2015). The astrocyte and microglial responses may have dual and opposing effects on the CNS. These cells can be neuroprotective because they secrete several neurotrophic factors and remove toxins (Gonzalez-Perez et al., 2015). However, these cells can also exert a neurotoxic effect because they secrete inflammatory cytokines and produce nitric oxide and other reactive oxygen species (ROS) that lead to neuronal damage and cell death (Gonzalez-Perez et al., 2012).

The molecular mechanisms that underlie the cytoarchitectural alterations in the brain of solvent users are unclear. However, a recent study found that cyclohexane promotes the overexpression of AP endonuclease 1 (APE1) in the hippocampus. This protein activates the cellular response to oxidative stress and regulates the transcription of genes involved in neuronal survival and DNA repair (Campos-Ordonez et al., 2015). This suggests that cyclohexane perturbs the redox balance in cells and affects the ability of tissue to detoxify ROS. The accumulation of ROS causes cellular dysfunction by damaging membranes, lipids, proteins, mitochondria, and DNA. However, additional studies are needed to clarify the role of ROS in cyclohexane-induced neurodegeneration.

The growing use of cyclohexane as a relatively safe replacement for benzene or toluene in a myriad of commercial products, including electronic cigarettes, necessitates a better understanding of the biological effects of this solvent. Insight into the cellular and molecular mechanisms of neural degeneration induced by cyclohexane will help minimize the potential risk associated with the intentional or accidental inhalation of this volatile compound.

## Author contributions

TC: Work conception and manuscript writing. OG: Work conception, manuscript writing and financing.

### Conflict of interest statement

The authors declare that the research was conducted in the absence of any commercial or financial relationships that could be construed as a potential conflict of interest.

## References

[B1] Bespalov A. Sukhotina I. Medvedev I. Malyshkin A. Belozertseva I. Balster R.. (2003). Facilitation of electrical brain self-stimulation behavior by abused solvents. Pharmacol. Biochem. Behav. 75, 199–208. 10.1016/S0091-3057(03)00071-6 12759128

[B2] Bowen S. E. Batis J. C. Paez-Martinez N. Cruz S. L. (2006). The last decade of solvent research in animal models of abuse: mechanistic and behavioral studies. Neurotoxicol. Teratol. 28, 636–647. 10.1016/j.ntt.2006.09.005 17064879

[B3] Campos-Ordonez T. Zarate-Lopez D. Galvez-Contreras A. Y. Moy-Lopez N. Guzman-Muniz J. Gonzalez-Perez O. (2015). Cyclohexane produces behavioral deficits associated with astrogliosis and microglial reactivity in the adult hippocampus mouse brain. Cell. Mol. Neurobiol. 35, 503–512. 10.1007/s10571-014-0146-6 25433657 PMC11486179

[B4] Fan Z. Heng Y. Hua-Dong Z. Yan-Jiang W. (2014). Toluene-induced leukoencephalopathy with characteristic magnetic resonance imaging findings. Neuroimmunol. Neuroinflamm. 1, 92–94. 10.4103/2347-8659.139721

[B5] Gonzalez-Perez O. Gutierrez-Fernandez F. Lopez-Virgen V. Collas-Aguilar J. Quinones-Hinojosa A. Garcia-Verdugo J. M. (2012). Immunological regulation of neurogenic niches in the adult brain. Neuroscience 226, 270–281. 10.1016/j.neuroscience.2012.08.053 22986164 PMC3490038

[B6] Gonzalez-Perez O. Lopez-Virgen V. Quinones-Hinojosa A. (2015). Astrocytes: everything but the glue. Neuroimmunol. Neuroinflamm. 2, 115–117. 10.4103/2347-8659.153979 25938129 PMC4414035

[B7] Johnston L. O'malley P. Miech R. Bachman J. Schulenberg J. (2014). Monitoring the Future National Results on Drug Use: 1975–2013: Overview, Key Findings on Adolescent Drug Use. Ann Arbor: Institute for Social Research, The University of Michigan.

[B8] Lammers J. H. Emmen H. H. Muijser H. Hoogendijk E. M. Mckee R. H. Owen D. E.. (2009). Neurobehavioral effects of cyclohexane in rat and human. Int. J. Toxicol. 28, 488–497. 10.1177/1091581809345534 19966141

[B9] Mutti A. Cavatorta A. Lucertini S. Arfini G. Falzoi M. Franchini I. (1982). Neurophysiological changes in workers exposed to organic solvents in a shoe factory. Scand. J. Work Environ. Health 8(Suppl. 1), 136–141. 7100840

[B10] Ramcharan K. Ramesar A. Ramdath M. Teelucksingh J. Gosein M. (2014). Encephalopathy and neuropathy due to glue, paint thinner, and gasoline sniffing in Trinidad and Tobago-MRI findings. Case Rep. Neurol. Med. 2014, 850109. 10.1155/2014/850109 25045557 PMC4087279

[B11] Ridenour T. A. Bray B. C. Cottler L. B. (2007). Reliability of use, abuse, and dependence of four types of inhalants in adolescents and young adults. Drug Alcohol Depend. 91, 40–49. 10.1016/j.drugalcdep.2007.05.004 17576041 PMC2040516

[B12] Sikkema J. De Bont J. A. Poolman B. (1995). Mechanisms of membrane toxicity of hydrocarbons. Microbiol. Rev. 59, 201–222. 7603409 10.1128/mr.59.2.201-222.1995 PMC239360

[B13] Yasugi T. Kawai T. Mizunuma K. Kishi R. Harabuchi I. Yuasa J.. (1994). Exposure monitoring and health effect studies of workers occupationally exposed to cyclohexane vapor. Int. Arch. Occup. Environ. Health 65, 343–350. 10.1007/BF00405700 8175191

[B14] Yuasa J. Kishi R. Eguchi T. Harabuchi I. Kawai T. Ikeda M.. (1996). Investigation on neurotoxicity of occupational exposure to cyclohexane: a neurophysiological study. Occup. Environ. Med. 53, 174–179. 10.1136/oem.53.3.174 8704858 PMC1128440

